# Supplementary material for: VRC01 Selects Rare HIV Escape Mutations After Acquisition in Antibody-Mediated Prevention Trials
Source: bioRxiv. 2025 Oct 29:2025.10.29.685411. Preprint. [Version 1] doi: 10.1101/2025.10.29.685411 (PMC12636421; doi:10.1101/2025.10.29.685411)
Supplement: Supplement 1 — Table S1. VRC01 Epitope amino acid positions Table S2. Metadata of participants with variant sites Table S3. Correlation coefficients calculated from random effects generalized linear model of Envelope backbones [file media-1.pdf]

Table S1. List of 34 amino acid positions/sites within the VRC01 epitope that were evaluated for changes over time.

HXB2 amino acid position/site	Feature	Envelope Domain	Experiment	References/Citations
197	Glycosylation site	Glycosylation site	Neutralization	1
198	Glycosylation site	CD4 contact	Neutralization	1
230	Glycosylation site	Glycosylation site, Sieve site	Sieve analysis	2
276	VRC01 contact site	Loop D	Neutralization	3
278	VRC01 contact site	Loop D	Neutralization	4
279	VRC01 contact site	Loop D (CD4 contact)	Neutralization	3
280	VRC01 contact site	Loop D (CD4 contact)	Neutralization	4
281	VRC01 contact site	Loop D (CD4 contact)	Neutralization	4
282	VRC01 contact site	Loop D (CD4 contact)	Neutralization	6
365	VRC01 contact site	CD4 Binding loop	Neutralization	7
366	VRC01 contact site	CD4 Binding loop	Neutralization	6
367	VRC01 contact site	CD4 Binding loop	Neutralization	6
368	VRC01 contact site	CD4 Binding loop	Neutralization	6
369	VRC01 contact site	CD4 Binding loop	Neutralization	1
370	VRC01 contact site	CD4 Binding loop	Neutralization	6
371	VRC01 contact site	CD4 Binding loop	Neutralization	6
427	VRC01 contact site	β20, β21 (CD4 contacts)	Binding	1
428	VRC01 contact site	β20, β21 (CD4 contacts)	Binding	1
430	VRC01 contact site	β20, β21 (CD4 contacts)	Binding	1
455	VRC01 contact site	β23 (CD4 contacts)	Neutralization	7
456	VRC01 contact site	β23 (CD4 contacts)	Neutralization	8
457	VRC01 contact site	β23 (CD4 contacts)	Neutralization	6
458	VRC01 contact site	β23 (CD4 contacts)	Binding	1
459	VRC01 contact site	CD4 contact	Neutralization	7
460	VRC01 contact site	V5 Loop (CD4 contact)	Neutralization	4
461	VRC01 contact site	V5 Loop (CD4 contact)	Neutralization	3
462	VRC01 contact site	V5 Loop	Binding	1
463	VRC01 contact site	V5 Loop	Neutralization	3
465	VRC01 contact site	V5 Loop β24	Binding	1
469	VRC01 contact site	V5 Loop β24 (CD4 contact)	Binding	1
471	Glycosylation site	β24 (CD4 contact)	Neutralization	4
472	VRC01 contact site	CD4 contact	Binding	9
473	VRC01 contact site	CD4 contact	Neutralization	7
474	VRC01 contact site	CD4 contact	Neutralization	6

References/Citations

[1]Dingens, et al.2019. Immunity, Volume 50, Issue 2, 520 - 532.e3
[2]Juraska, et al., 2024, PNAS 121: e2308942121
[3]Falkowska, et al. J Virol. 2012;86(8):4394-4403
[4]LaBranche, et al. 2019 PLOS Pathog 14(11): e1007646
[5]Schommers, et al. Cell. 2020;180(3):471-489.e22
[6]Huang, et al. Immunity. 2016;45(5):1108-1121
[7]Cheng, et al. JCI Insight. 2018;3(5):e97018
[8]Foulkes, et al. PLoS Pathog. 2025;21(1):e1012825.
[9]Zhou, et al. Science. 2010;329(5993):811-817

Table S2. Metadata of participants with variant sites

Trial	PtID	Treatment Arm	Parental (WT)/Mutation	VRC01 Sensitive/ Resistant	VRC01 infusion to TP1 (days)	TP1-TP2 (days)	No. Sequences Time point 1	No. Sequences Time point 2
703	0520	10 mg/kg	WT	Sensitive	0	28	84	291
			D474N	Sensitive			4	2
703	0578	10 mg/kg	WT	Sensitive	0	9	137	153
			N280D	Resistant			25	131
			G458E	Resistant			7	22
			N280D, G458E	Resistant			0	1
703	0597	10 mg/kg	WT	Sensitive	0	6	215	115
			S461N	Sensitive			89	62
703	0860	10 mg/kg	WT	Sensitive	28	14	171	121
			N276T	Resistant			0	4
			N276I	Resistant			1	2
703	0967	10 mg/kg	WT	Sensitive	81	8	42	23
			N280D	Resistant			0	5
703	1551	10 mg/kg	WT	Sensitive	177	17	36	339
			S365P	Sensitive			0	13
703	1750	Placebo	WT	Sensitive	No infusion	8	252	361
			V455I	Sensitive			0	7
703	1783	10 mg/kg	WT	Sensitive	0	9	361	393
			N197T	Resistant			0	17
			D279A	Resistant			0	4
			K460N	Resistant			0	26
			N197T, K460N	Resistant			0	1
703	2141	10 mg/kg	WT	Sensitive	0	26	107	203
			D279G	Resistant			0	6
703	2372	10 mg/kg	WT	Sensitive	0	23	374	192
			D279G	Resistant			0	91
			D279Y	Resistant			0	4
			D279A	Resistant			0	3
			N280D	Resistant			0	26
			G459D	Resistant			0	26
			D279A, G459D	Resistant			0	1
			N280D, G459D	Resistant			0	1
703	2631	Placebo	WT	Sensitive	No infusion	15	95	132
			N461K	Sensitive			2	5
704	0601	10 mg/kg	WT	Sensitive	0	28	123	89
			N279K	Resistant			0	50
704	0855	Placebo	WT	Sensitive	No infusion	5	213	65
			E370K	N/A*			3	0
704	1481	10 mg/kg	WT	Sensitive	27	8	379	357
			D279Y	Resistant			93	190
704	1551	10 mg/kg	WT	Sensitive	0	6	182	30
			P369T	Sensitive			9	1
			P369S	Sensitive			1	0
			T455I	Sensitive			6	0
			T455A	Sensitive			1	1
			R456K	Sensitive			2	1
			D462Y	Sensitive			2	0
			D462G	Sensitive			1	0
704	1783	Placebo	WT	Sensitive	No infusion	20	395	264
			D461N	Sensitive			1	4
704	1802	10 mg/kg	WT	Sensitive	-27	27	297	90
			D474N	Sensitive			0	6
704	2063	Placebo	WT	Sensitive	No infusion	28	297	90
			G459E	Sensitive			0	7

*Pseudoviruses with E370K were not functional.

PtID, Participant study identifier, WT - wildtype

Table S3: Correlation co-efficients calculated from random effects generalized linear model of Envelope backbones

bNAbs		Corr. Coeff. (95% CI)	Pvalue
VRC01	VRC01.23LS	0.95 (0.74, 1)	9.7E-11
	VRC07-523LS	0.84 (0.56, 1)	2.3E-07
	3BNC117	1 (0.86, 1)	1.9E-14
	N6	0.32 (0.05, 0.59)	0.02
	1-18	-0.12 (-0.36, 0.14)	0.36
	CH235.12	0.13 (-0.19, 0.47)	0.41
	VRC_CH31	0.96 (0.79, 1)	1.3E-15
	PG9	0.09 (-0.17, 0.35)	0.49
	10E8.v4	-0.21 (-0.47, 0.05)	0.11
VRC01.23LS	VRC07-523LS	0.66 (0.48, 0.85)	1.9E-09
	3BNC117	0.49 (0.26, 0.76)	8.5E-05
	N6	0.29 (0.12, 0.48)	1.8E-03
	1-18	0.1 (-0.06, 0.28)	0.21
	CH235.12	0.24 (0.02, 0.46)	0.03
	VRC_CH31	0.5 (0.31, 0.72)	2.4E-06
	PG9	0.05 (-0.12, 0.23)	0.52
	10E8.v4	-0.09 (-0.27, 0.09)	0.32
VRC07-523LS	3BNC117	0.54 (0.3, 0.79)	3.8E-05
	N6	0.49 (0.33, 0.65)	1.0E-07
	1-18	0.08 (-0.1, 0.28)	0.37
	CH235.12	0.24 (-0.01, 0.51)	0.06
	VRC_CH31	0.47 (0.27, 0.66)	1.4E-05
	PG9	0.04 (-0.14, 0.24)	0.63
	10E8.v4	0.01 (-0.18, 0.22)	0.87
3BNC117	N6	0.12 (-0.1, 0.36)	0.28
	1-18	-0.18 (-0.36, 0.03)	0.09
	CH235.12	0.16 (-0.09, 0.42)	0.21
	VRC_CH31	0.76 (0.63, 0.88)	2.1E-17
	PG9	0.05 (-0.15, 0.25)	0.62
	10E8.v4	-0.16 (-0.36, 0.05)	0.13
N6	1-18	0.45 (0.23, 0.71)	1.7E-04
	CH235.12	0.49 (0.12, 0.97)	0.01
	VRC_CH31	0.28 (-0.02, 0.58)	0.06
	PG9	0.12 (-0.14, 0.39)	0.36
	10E8.v4	0.22 (-0.04, 0.5)	0.10
1-18	CH235.12	0.65 (0.38, 0.92)	3.9E-05
	VRC_CH31	0.1 (-0.23, 0.42)	0.46
	PG9	0.2 (-0.09, 0.5)	0.17
	10E8.v4	0.41 (0.15, 0.69)	3.3E-03
CH235.12	VRC_CH31	0.2 (-0.05, 0.45)	0.11
	PG9	0.16 (-0.06, 0.4)	0.16
	10E8.v4	0.27 (0.03, 0.52)	0.03
VRC_CH31	PG9	0.07 (-0.15, 0.31)	0.51
	10E8.v4	-0.13 (-0.37, 0.11)	0.29
PG9	10E8.v4	0.55 (0.29, 0.78)	3.1E-05
